# Supplementary material for: Myeloid apolipoprotein E controls dendritic cell antigen presentation and T cell activation
Source: Nat Commun. 2018 Aug 6;9:3083. doi: 10.1038/s41467-018-05322-1 (PMC6079066; doi:10.1038/s41467-018-05322-1)
Supplement: Supplementary file 1 — Supplementary Information [file 41467_2018_5322_MOESM1_ESM.pdf]

Myeloid apolipoprotein E controls dendritic cell antigen presentation and T cell activation

Bonacina et al.

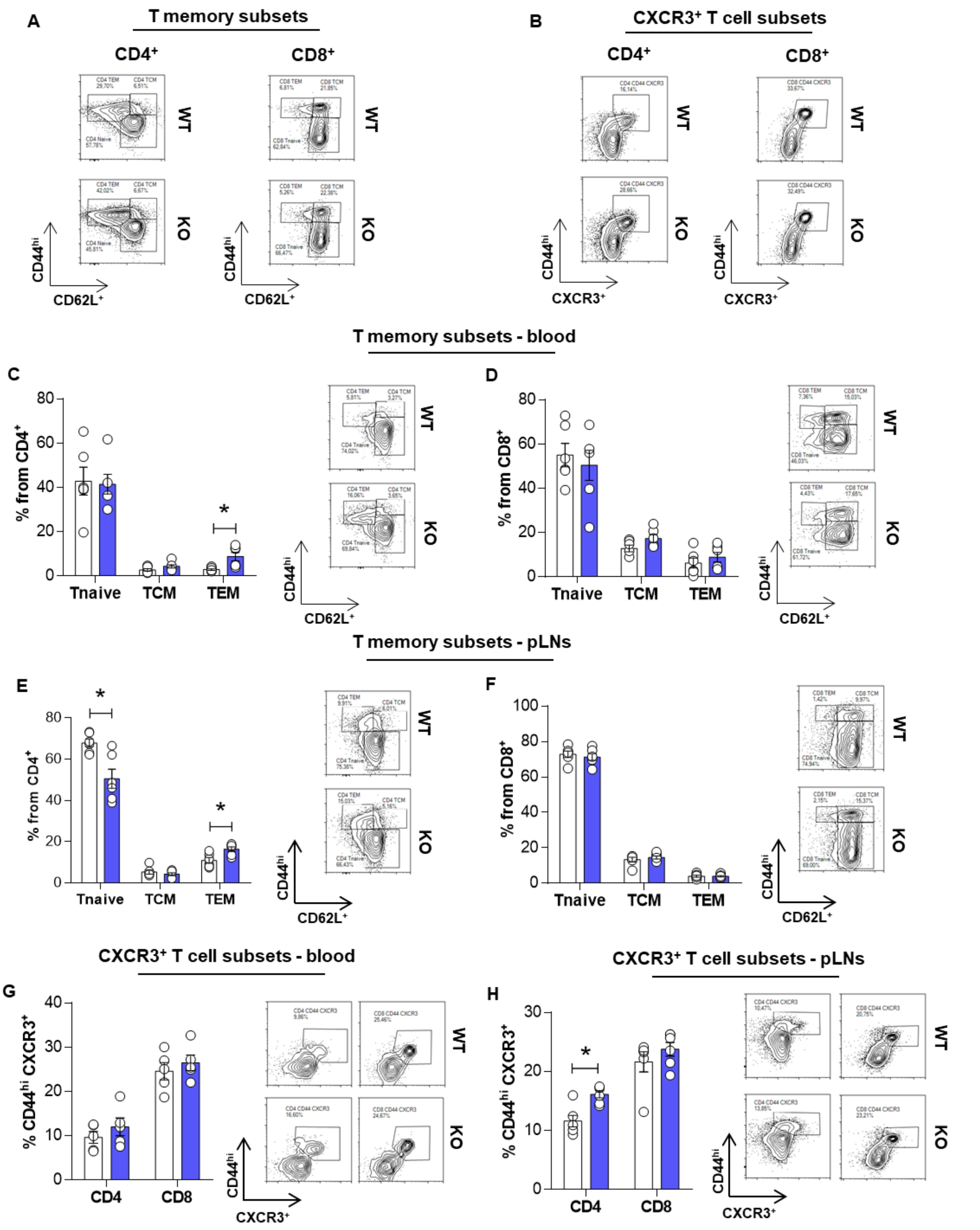

Supplementary Figure 1. Immunophenotyping of WT and apoE KO mice

A-B) Representative density plots of CD4$^+$ and CD8$^+$ T memory subsets (A) and activated CD44^{hi} CXCR3$^+$ (B) from the spleen of WT or apoE KO mice. **C-D**) Frequencies of CD4$^+$ (C) and CD8$^+$ (D) T

memory subsets in the blood of WT or apoE KO mice; representative pictures are shown. **E-F**) Frequencies of $CD4^+$ (E) and $CD8^+$ (F) T memory subsets in the peripheral lymph nodes of WT and apoE KO mice; representative pictures are shown. **G-H**) Frequencies of $CD4^+$ and $CD8^+$ activated $CD44^{hi}$ $CXCR3^+$ T cells in the blood (G) and peripheral lymph nodes (H) from WT and apoE KO mice. n=4-6 per group. Statistical analysis was performed with two-way Anova. Data are reported as mean ± SEM; *$p<0.05$.

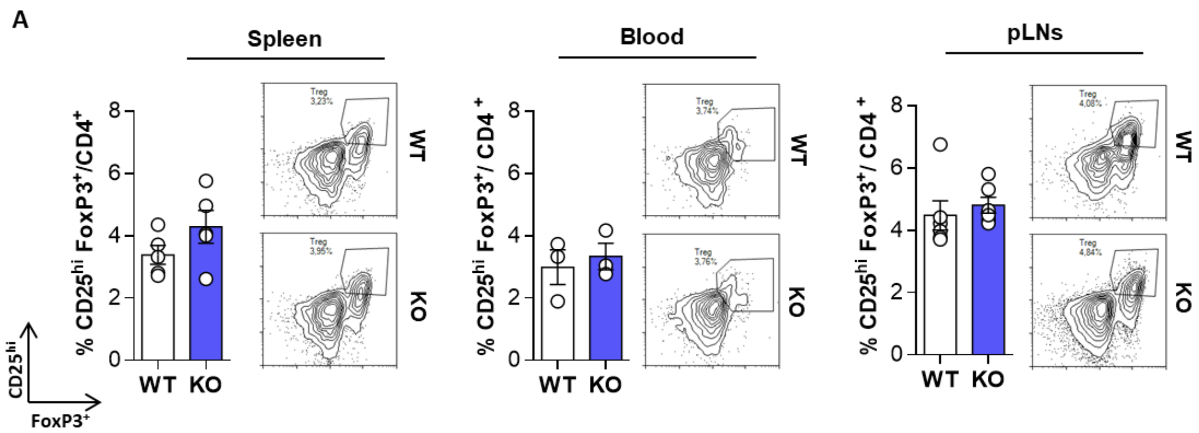

Supplementary Figure 2. ApoE KO mice show no difference in Treg frequency compared to WT mice

A) Frequency of Treg cells ($CD4^+CD25^{hi}FoxP3^+$) in the spleen, blood and peripheral lymph nodes of WT and apoE KO mice; representative dot plots are presented. n=3-6 per group. Statistical analysis was performed with unpaired Ttest. Data are reported as mean ± SEM.

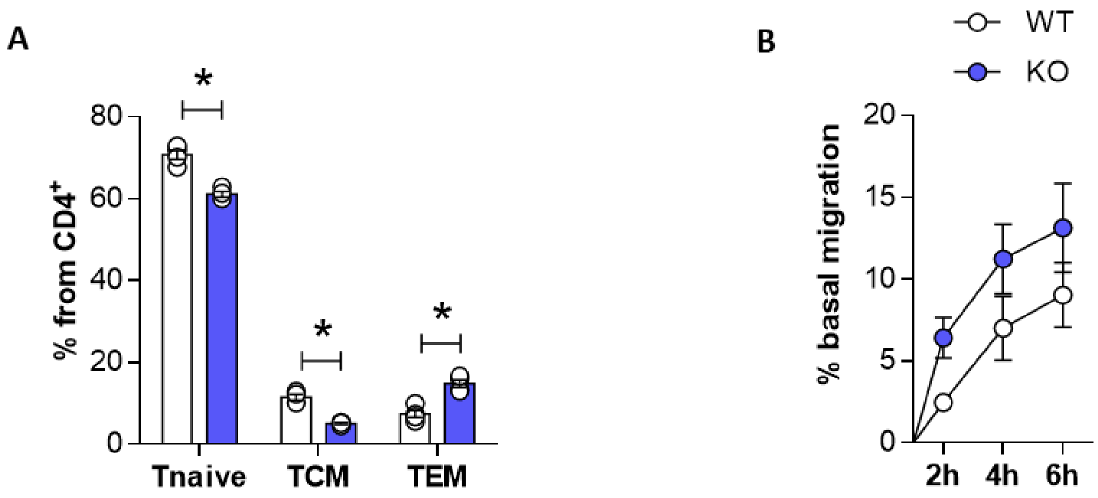

Supplementary Figure 3. Graft rejection is associated with the expansion of $CD4^+$ TEM in apoE KO mice

A) Frequency of $CD4^+$ T cells subsets in the draining lymph nodes of WT and apoE KO mice after skin allograft transplantation. B) Basal migratory response of lymphocytes isolated from draining lymph nodes after allograft rejection measured by transwell. n=4 per group. Statistical analysis was performed with two-way Anova. Data are reported as mean ± SEM; *$p<0.05$.

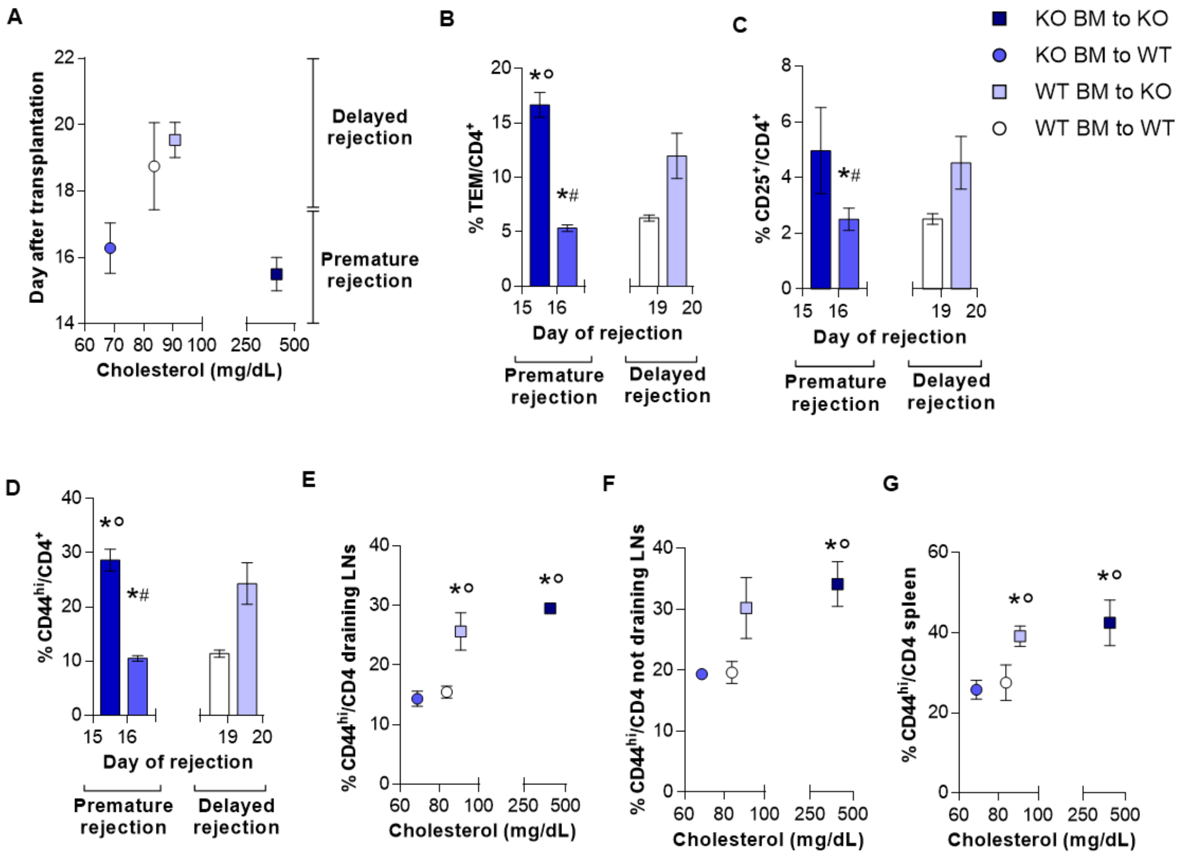

Supplementary Figure 4. Characterization of bone marrow transplanted WT and apoE KO mice

A) Plasma cholesterol levels (mg/dL) after allograft rejection presented compared to the mean of day rejection. **B-D)** Frequency of circulating $CD4^{+}T_{EM}$ (B), $CD4^{+}CD25^{+}$ (C) and $CD4^{+}CD44^{hi}$ (D) subsets presented as function of the mean of day rejection. **E-G)** Frequency of $CD4^{+}$ $CD44^{hi}$ subsets compared to plasma cholesterol levels (mg/dL) in draining (E), not draining (F) lymph nodes and spleen (G). N=4 per group. Statistical analysis was performed with two-way Anova. Data are reported as mean ± SEM; *p<0,05 vs WT BM to WT, ° vs KO BM to WT, # vs WT BM to KO.

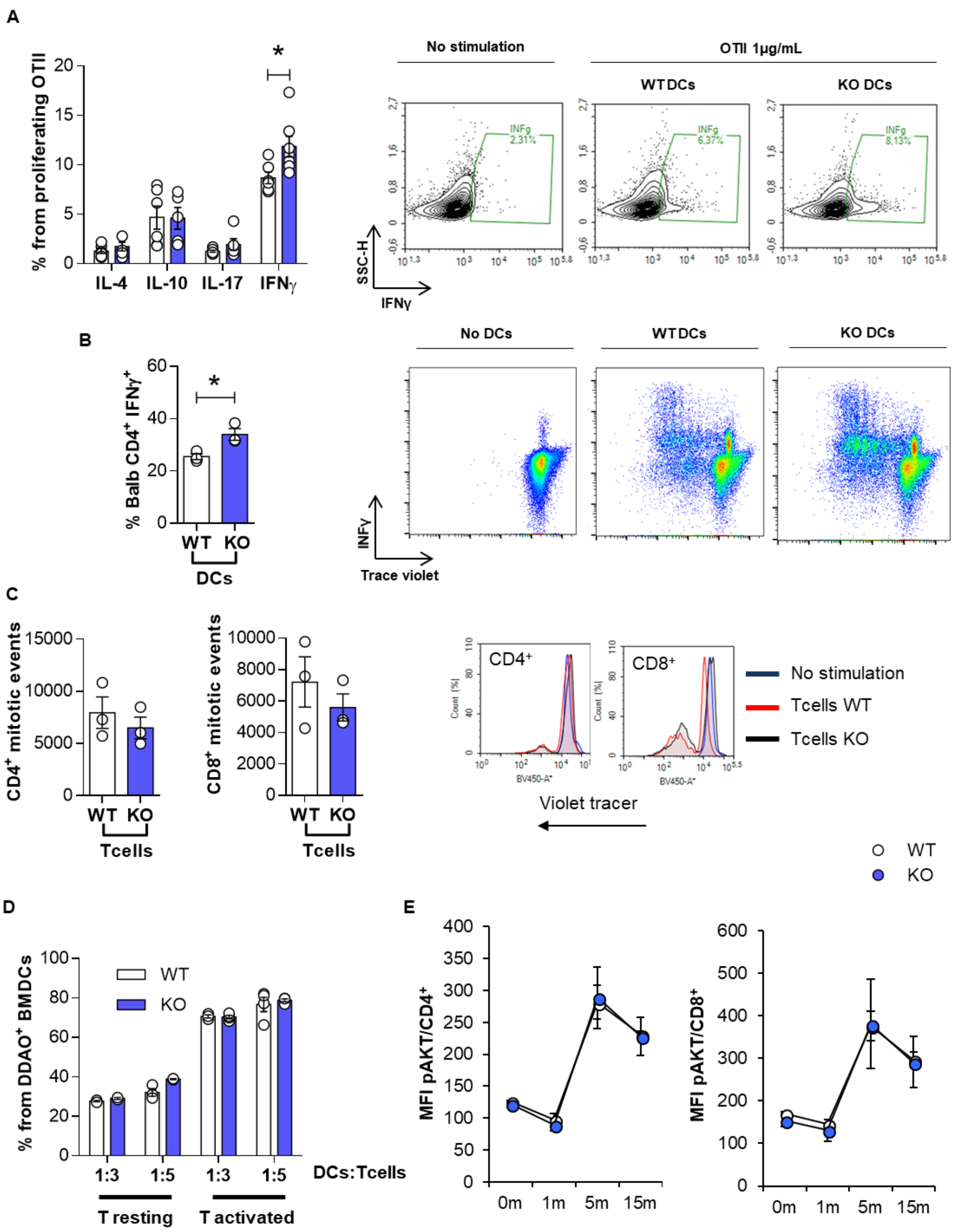

Supplementary Figure 5. T cell activation is not affected by apoE deficiency

A) Cytokine production (IL-4, IL-10, IL-17, IFNγ) by transgenic OT-II $CD4^+$ T cells co-cultured with WT or apoE KO BMDCs pulsed with OT-II peptide (10 μg/mL) for four days and re-challenged with OT-II peptide (1 μg/mL) for 4 hours; representative dot plots are shown. **B)** IFNγ production from BALB/c $CD4^+$ T cells co-cultured with WT or apoE KO spleen-derived DCs for five days;

representative dot plots are shown. **C)** Proliferation of $CD4^+$ and $CD8^+$ T cells isolated from WT and ApoE KO mice pulsed with allogenic BALB/c spleen-derived DCs for five days; representative histograms are shown. **D)** Conjugates between allogenic BALB/c BMDCs and resting or activated lymphocytes isolated from C57BL/6 WT and apoE KO analyzed by flow cytometry. **E)** Phosphorylation of AKT detected with flow cytometry in $CD4^+$ and $CD8^+$ T cells after *in vitro* anti-CD3/28 stimulation. N=3 (B-D in triplicate), N=6 (A) per group. Statistical analysis was performed with unpaired Ttest (A-C) and two-way Anova (D-E). Data are reported as mean ± SEM; $^*p<0.05$.

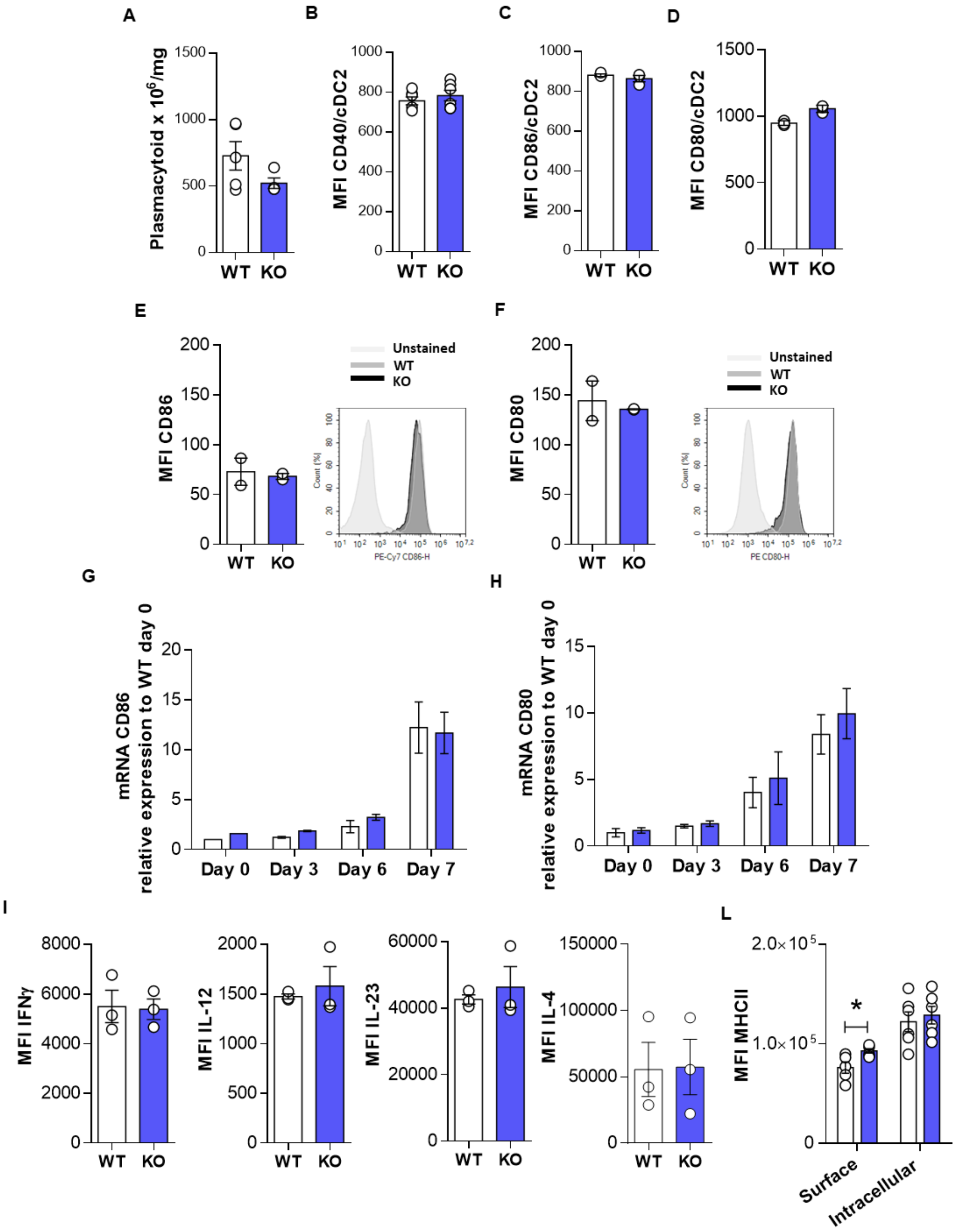

Supplementary Figure 6. Immunophenotyping of spleen-derived DCs and BMDCs

A) Number of plasmacytoid ($CD11c^{+}MHCII^{+}B220^{+}$) DCs corrected for spleen weight of WT or apoE KO mice. **B-D)** Mean fluorescence intensity (MFI) of cDC2 from WT and apoE KO mice for CD40 (B), CD86 (C) and CD80 (D). **E-F)** protein expression of CD86 (E) and CD80 (F) in BMDCs from WT and

apoE KO mice after stimulation with LPS (10 ng/mL) for 18h analysed by flow cytometry; representative histogram are shown. **G-H)** mRNA expression of CD86 (G) and CD80 (H) in BMDCs of WT and apoE KO mice before differentiation (day 0) and at 3, 6 and 7 days of differentiation and maturation (stimulation with LPS (10ng/mL) for 18h). **I**) mean fluorescence intensity (MFI) of IFNγ, IL-12, IL-23 and IL-10 in BMDCs after stimulation with LPS (10ng/mL) for 4h analysed by flow cytometry. **L)** mean fluorescence intensity (MFI) of MHCII in cDC2 from WT or apoE KO mice analysed as surface and intracellular expression by flow cytometry. N=2-5 per group. Statistical analysis was performed with unpaired Ttest. Data are reported as mean ± SEM; *p<0,05.

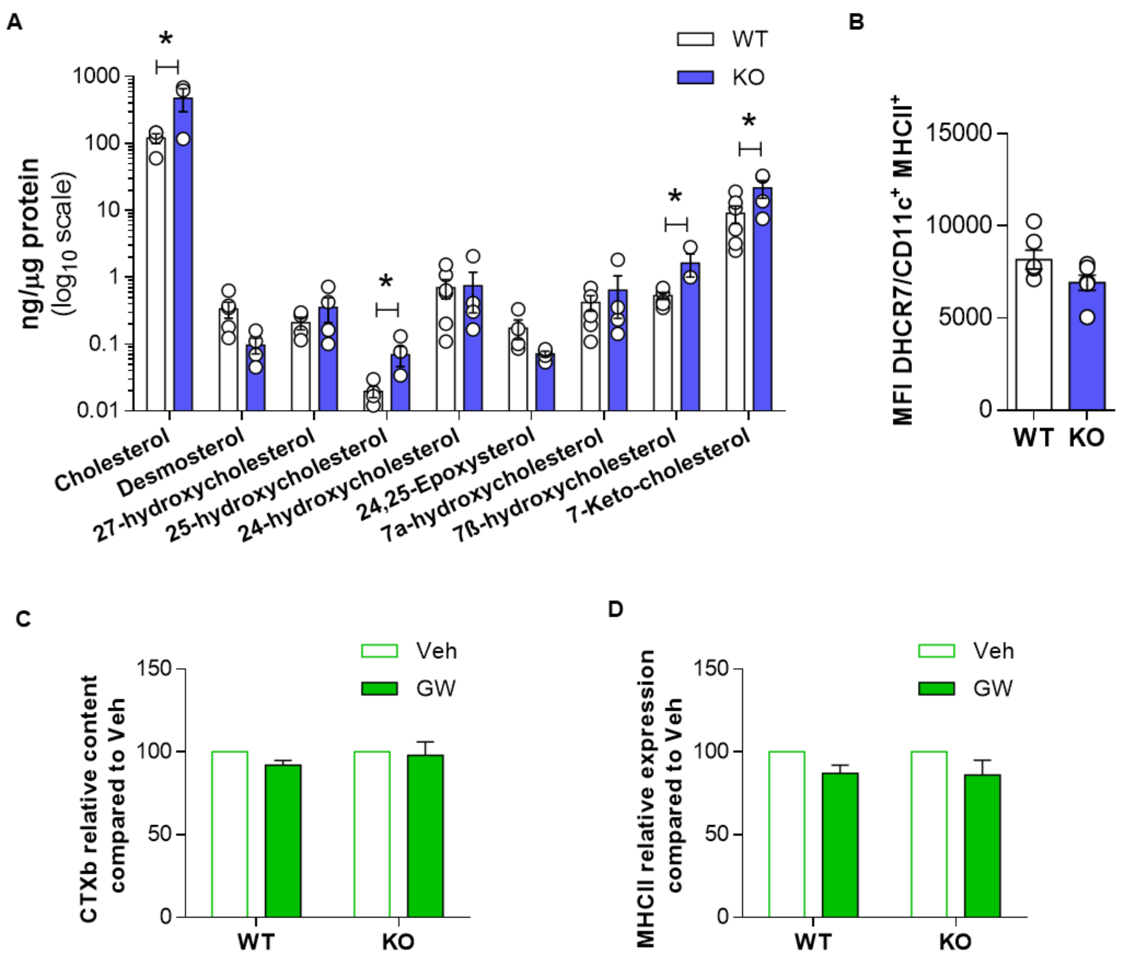

Supplementary Figure 7. Sterols and oxysterols profile of WT and apoE KO DCs

A) Determination (ng/µg) of sterols and oxysterols by gas chromatography-mass spectrometry of spleen-derived DCs. **B)** Mean fluorescence intensity (MFI) of DHCR7 in DCs of WT and apoE KO determined by facs analysis. **C-D)** lipid rafts content (CTXb staining, C) and MHCII expression (D) in mature BMDCs treated with the LXR agonist GW3965 (1 µM). n=4-6 per group. Statistical analysis was performed with unpaired Ttest. Data are reported as mean ± SEM; *p<0,05.

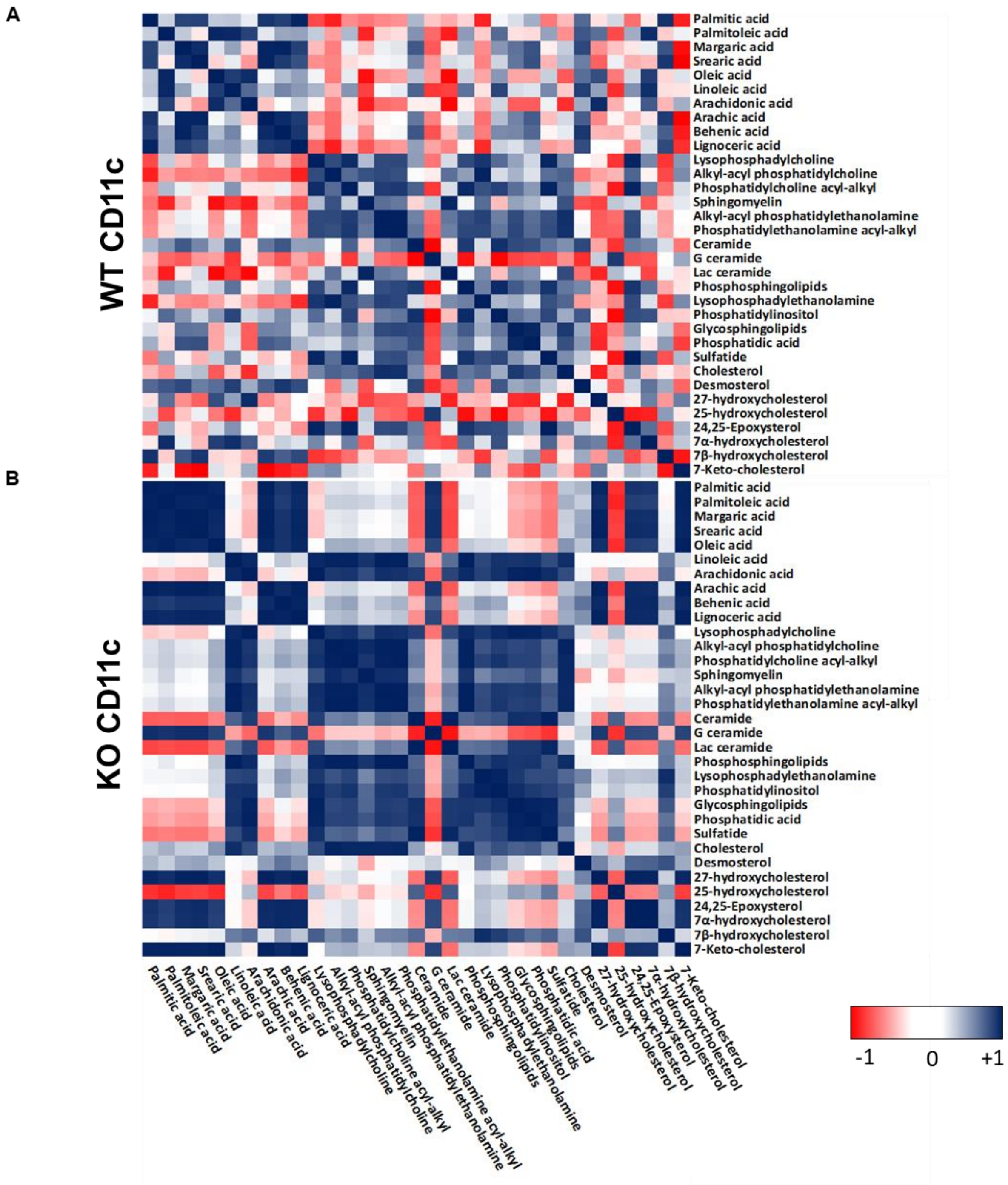

Supplementary Figure 8. Dependence between lipids of DCs of WT or apoE KO mice

A-B) Correlation matrix was used to investigate the dependence between phospholipids, fatty acids, sterols and oxysterols within CD11c^{+} isolated from the spleen of WT or apoE KO mice. Lipids were determined by gas chromatography-mass spectrometry. N=4 per group.

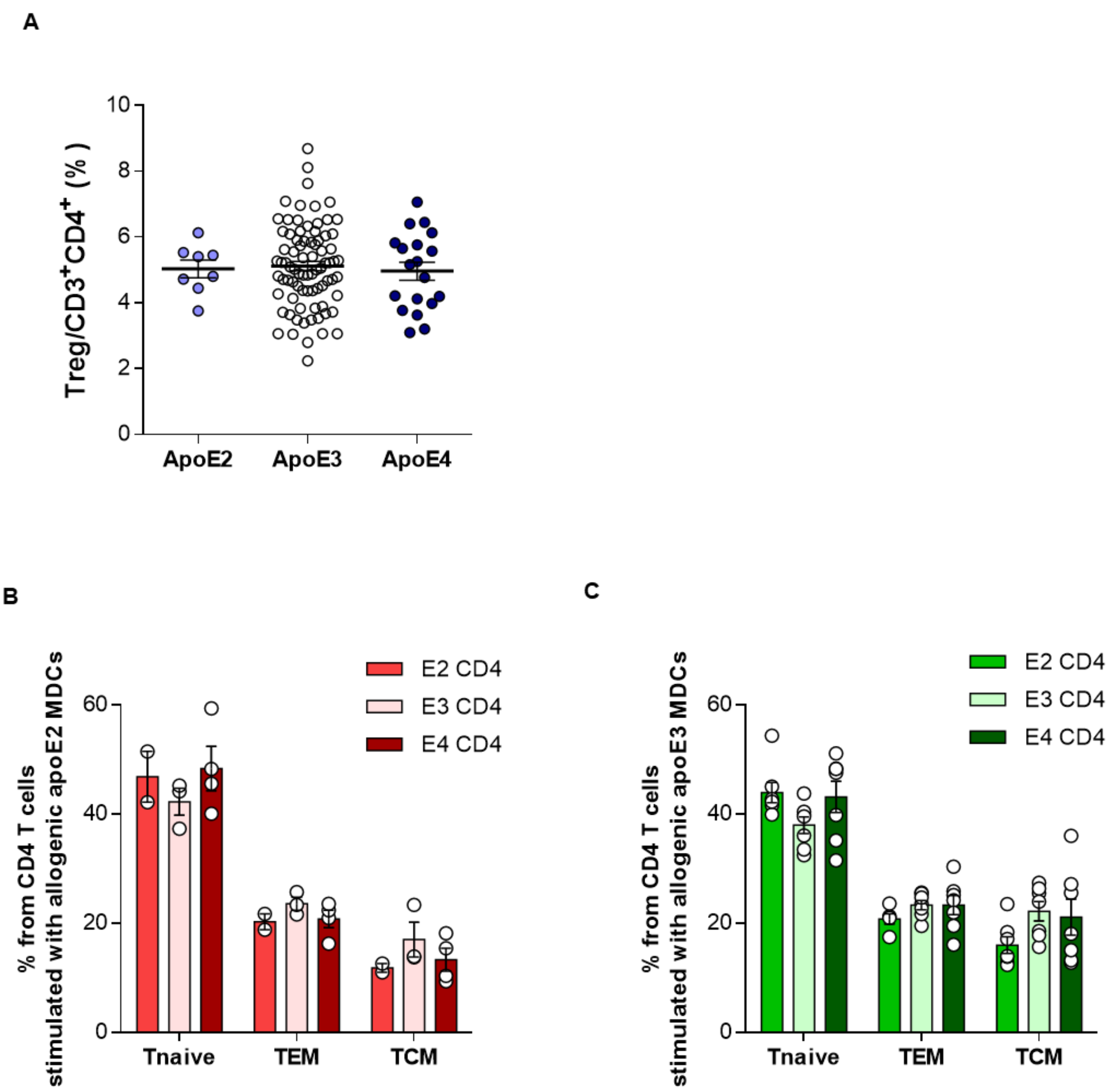

Supplementary Figure 9. Levels of circulating Treg cells in human carriers of different apoE isoforms and isoform-dependent MDCs reactivity tested with MLR assay

A) Levels of circulating Treg cells ($CD4^{+}CD25^{hi}CD127^{lo}$) in human carriers of apoE isoforms. **B-C)** Polarization of $CD4^{+}$ T_{naive} cells from carriers of apoE2, apoE3 or apoE4 isoform with allogenic MDCs from apoE2 (B) and apoE3 (C) carriers. N=2-5 per group (B-C). Statistical analysis was performed with two-way Anova. Data are reported as mean ± SEM.

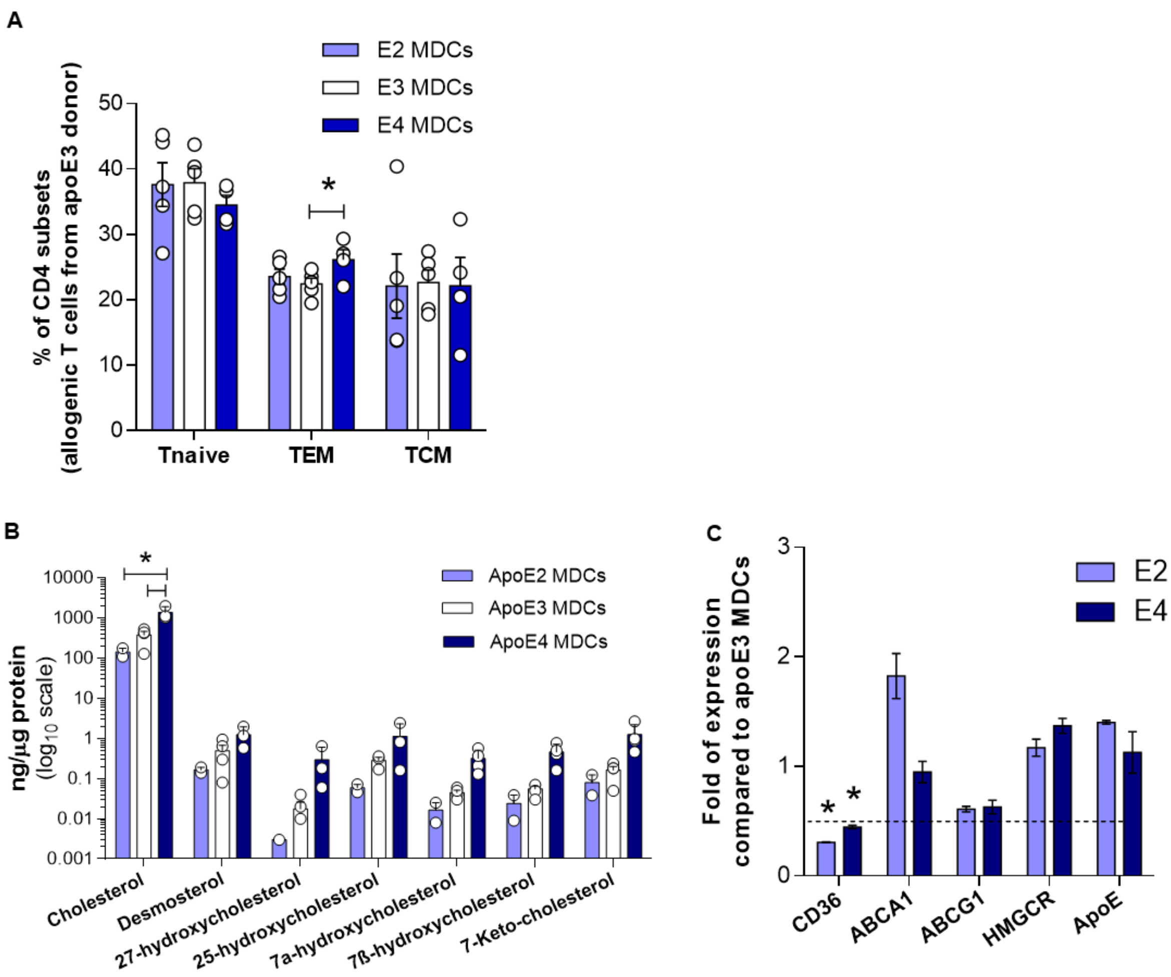

Supplementary Figure 10. ApoE isoform-dependent T cell polarization and MDCs reactivity tested with MLR assay

A) Polarization of CD4+ T_{naive} cells from a carrier of apoE3 isoform with allogenic MDCs from apoE2, apoE3 and apoE4 carriers. **B)** Determination (ng/µg) of sterols and oxysterols by gas chromatography-mass spectrometry of MDCs from apoE2, apoE3 and apoE4 carriers. **C)** Expression of CD36, ABCA1, ABCG1, HMGCR and ApoE mRNA by MDCs from apoE2 and apoE4 carriers compared to apoE3 carriers. N=2-4 per group. Statistical analysis was performed with two-way Anova. Data are reported as mean ± SEM; *$p<0.05$.

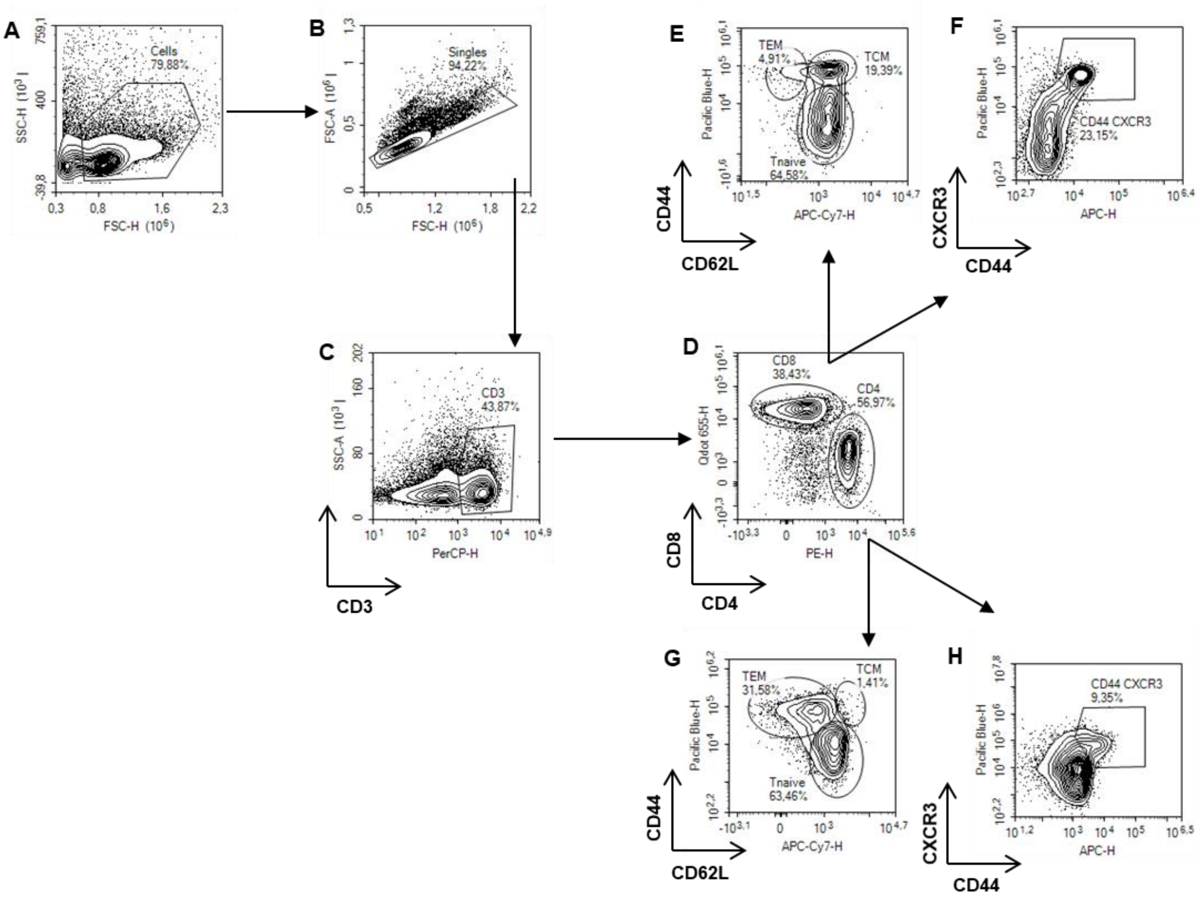

Supplementary Figure 11. Gating strategy for murine T cell subset analysis in lymph nodes and spleen

A) Live cells were gated based on dimension (FSC-H vs SSC-H) and single cells discriminated (**B**). T cells were selected on CD3 positivity (**C**) and further discriminated in $CD4^+$ and $CD8^+$ T cells (**D**). $CD4^+$ and $CD8^+$ T subsets were identified in T_{naive} ($CD62L^+$ $CD44^-$), T central memory (T_{CM}, $CD62L^+$ $CD44^+$) and T effector memory (T_{EM}, $CD62L^-$ $CD44^+$) (**E**, **G**) and activated T cell ($CD44^{hi}$ $CXCR3^+$) (**F**, **H**).

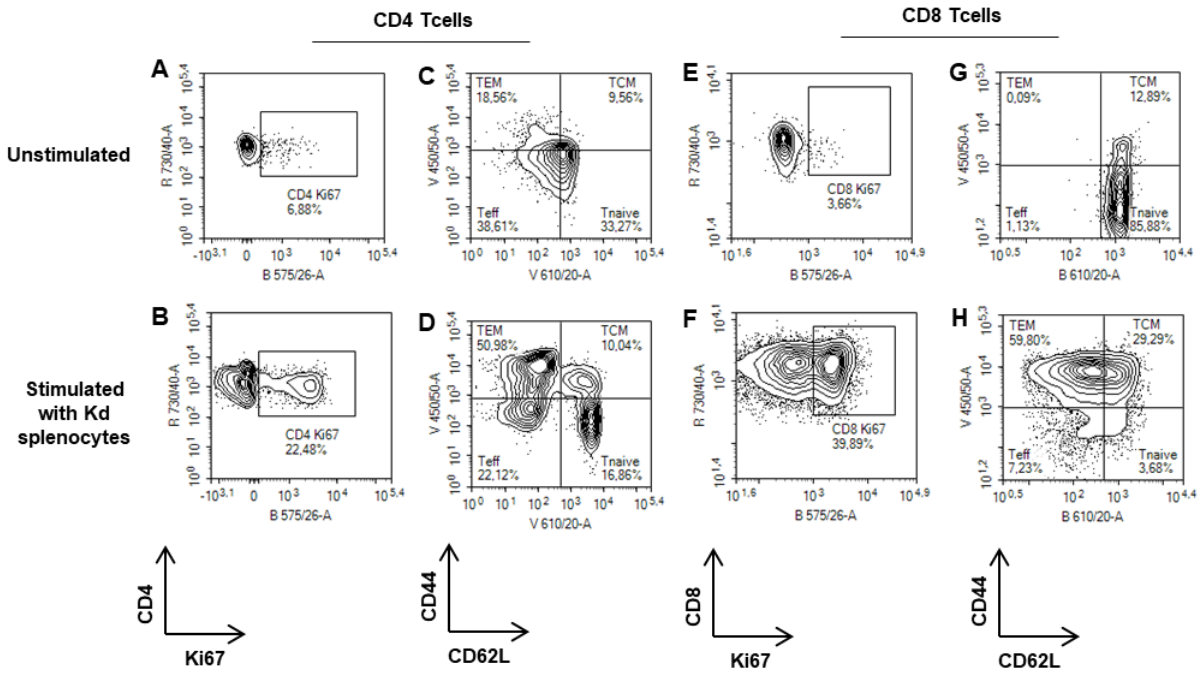

Supplementary Figure 12. Gating strategy for analysis of T cells from grafted WT and apoE KO BMT mice after challenge with K^d splenocytes

A-B) Proliferating $CD4^+$ T cells were identified by Ki67 staining; (**C-D**) CD4 polarization was analyzed with CD44 and CD62L staining and identified as T_{naive} ($CD62L^+$ $CD44^-$), T central memory (T_{CM}, $CD62L^+$ $CD44^+$) and T effector memory (T_{EM}, $CD62L^-$ $CD44^+$); (**E-F**) Proliferating $CD8^+$ T cells were identified by Ki67 staining; (**G-H**) CD8 polarization was analyzed with CD44 and CD62L staining and identified as T_{naive} ($CD62L^+$ $CD44^-$), T central memory (T_{CM}, $CD62L^+$ $CD44^+$) and T effector memory (T_{EM}, $CD62L^-$ $CD44^+$).

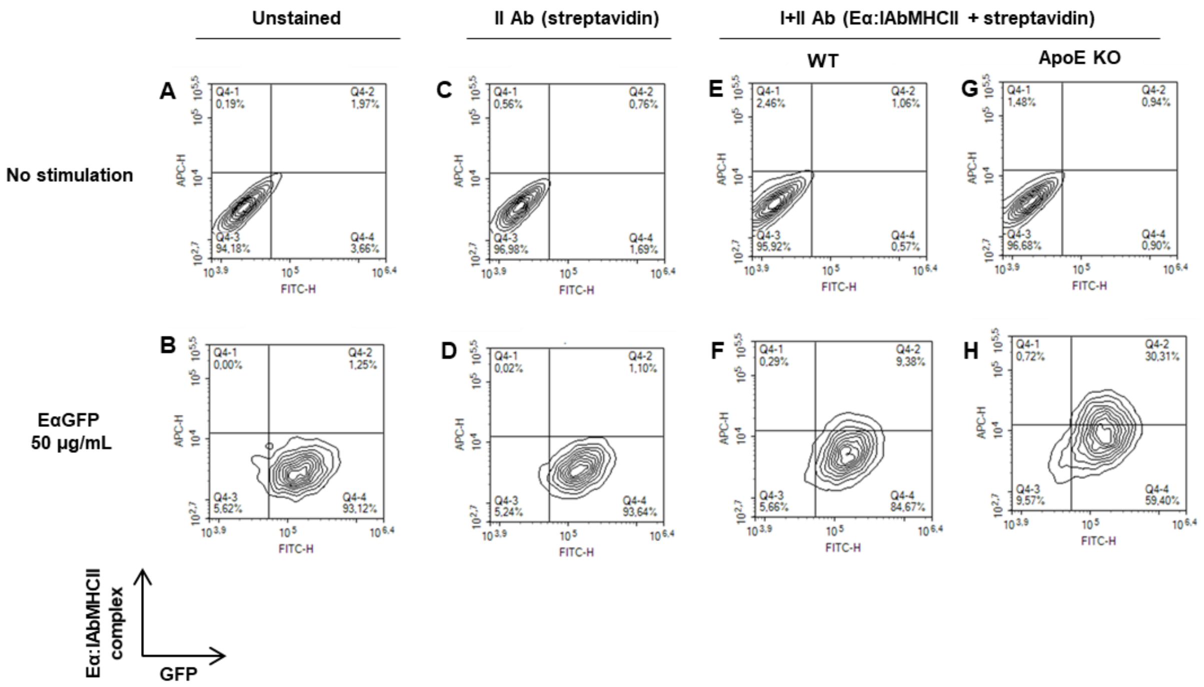

Supplementary Figure 13. Control plots for EαGFP uptake and presentation in BMDCs from WT and apoE KO mice.

BMDCs were treated or not with EαGFP 50 µg/mL for 4 hours; gating of BMDCs unstained (**A-B**), stained with the secondary antibody conjugated with streptavidin and the APC fluorochrome (**C-D**) and with the primary antibody against the Eα:IAbMHCII complex and streptavidin (**E-H**) are shown.

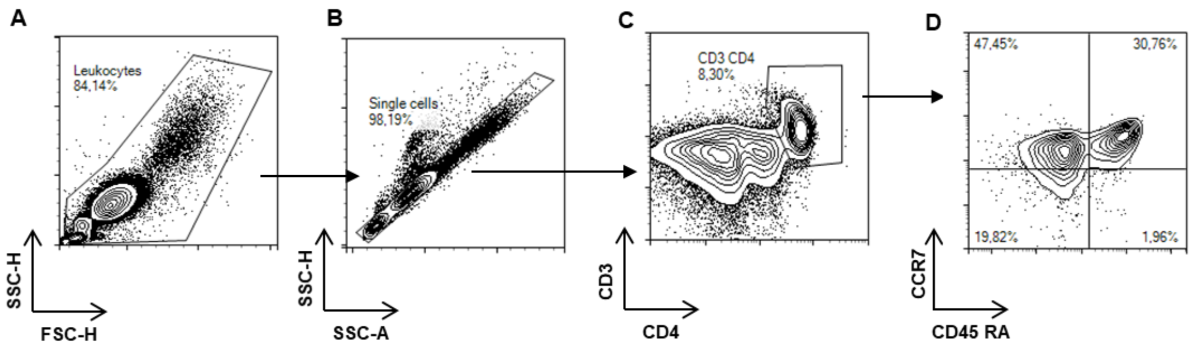

Supplementary Figure 14. Gating strategy for the analysis of T cells from human blood

A) Circulating leukocytes were gated based on dimension (FSC-H vs SSC-H) and single cells discriminated (B). $CD4^+$ T cells were selected depending on CD3 and CD4 positivity (C) and further discriminated in T_{naive} ($CD45RA^+$ $CCR7^+$), T central memory (T_{CM}, $CD45RA^-$ $CCR7^+$) and T effector memory (T_{EM}, $CD45RA^-$ $CCR7^-$) (D).

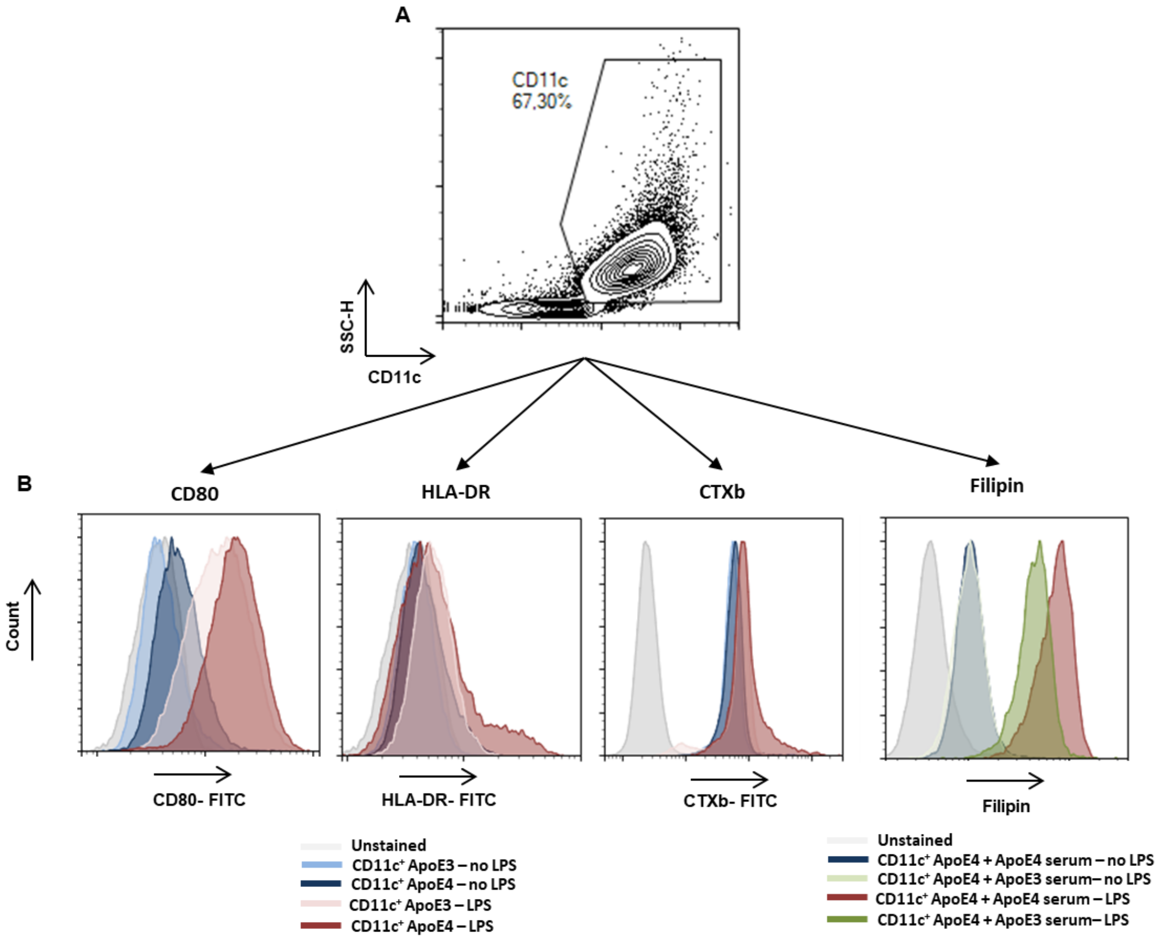

Supplementary Figure 15. Representative histograms, gating strategy and FMO controls of MDCs characterization

A) MDCs were discriminated based on CD11c positivity and the Median Fluorescence Intensity (MFI) of CD80, HLA-DR, CTXb (cholera toxin subunit B) and filipin was evaluated (B).

Supplementary Table 1. Clinical Characteristics, Biological Parameters and Therapies of Carriers of apoE isoforms From the General Population (PLIC Study)

	ApoE 2/3 (n=14)	ApoE 3/3 (n=121)	ApoE 3/4 (n=23)	*p*value
Age (years)	53 (14)	56 (13)	51 (13)	0.213
Gender (n, male)	7	58	10	0.908
BMI (Kg/m^2)	25.40 (3.44)	26.17 (3.75)	27.05 (3.76)	0.513
Waist/hip circumferences ratio	0.856 (0.074)	0.879 (0.094)	0.878 (0.084)	0.605
Systolic blood pressure (mmHg)	133 (8)	124 (17)	122 (15)	0.163
Diastolic blood pressure (mmHg)	78 (5)	76 (9)	72 (9)	0.124
Diagnosis of hyperthension (n, yes)	5	38	4	0.295
Anti-hyperensive (n, yes)	4	36	3	0.244
Fasting glucose levels (mg/dL)	102.71 (24.96)	101.30 (21.69)	100.39 (19.15)	0.290
Diagnosis of type 2 Diabetes (n, yes)	0	11	2	0.541
Glucose lowering drugs (n, yes)	0	3	1	0.727
Total cholestrol (mg/dL)	196.14 (35.81)	221.00 (40.51)	225.82 (40.24)	0.065
HDL-C (mg/dL)	62.50 (10.95)	56.31 (13.14)	56.60 (11.75)	0.077
Triglycerides (mg/dL)	85.35 (23.12)	103.55 (58.23)	100.60 (45.80)	0.623
LDL-C (Friedewald formula)	116.60 (38.59)	144.31 (35.26)	153.10 (40.15)	**0.043 (*, **)**
ApoB (mg/dL)	101.79 (18.21)	114.11 (25.59)	117.87 (32.56)	0.186
Lipid lowering drugs (n, yes)	2	21	2	0.591
Alanine Amino-Transferase (ALT, U/l)	24.78 (7.80)	26.67 (13.10)	23.34 (12.62)	0.425
Aspartate Amino-Transferase (AST, U/l)	23.50 (8.53)	22.59 (8.26)	20.17 (5.13)	0.334
Glutamyl Transferase (GGT, U/l)	36.92 (10.56)	35.47 (3.25)	25.91 (2.60)	0.370

Table legend. Data are presented as mean±standard deviation. BMI indicates body mass index; HDL-C, high-density lipoprotein cholesterol; LDL-C, low-density lipoprotein cholesterol.

* E2 vs E3; ** E2 vs E4

Supplementary Table 2. List of antibodies used in the paper.

ANTIBODIES	SOURCE	IDENTIFIER
Mouse monoclonal anti-CD3e PerCP Cy5.5	eBioscience	Cat#45-0031<br>RRID:AB_1107000
Mouse monoclonal anti-CD4 PECF594	BD Biosciences	Cat#562285<br>RRID:AB_11154410
Mouse monoclonal anti-CD4 FITC	eBioscience	Cat#11-0042-86<br>RRID:AB_464898
Mouse monoclonal anti-CD8 eVolve™ 655	eBioscience	Cat#86-0081<br>RRID:AB_2574783
Mouse monoclonal anti-CD8a FITC	eBioscience	Cat#11-0081<br>RRID:AB_464916
Mouse monoclonal anti-CD8a PerCP Cy 5.5	eBioscience	Cat#:45-0081-82<br>RRID:AB_1107004
Mouse monoclonal anti-CD44 eFluor® 450	eBioscience	Cat#48-0441<br>RRID:AB_1272246
Mouse monoclonal anti-CD62L APC-eFluor® 780	eBioscience	Cat#47-0621<br>RRID:AB_1603256
Mouse monoclonal anti-CD25 PE/Cy7	eBioscience	Cat#25-0251-82<br>RRID:AB_469608
Mouse monoclonal anti-CD183 (CXCR3) APC	eBioscience	Cat#17-1831-80<br>RRID:AB_1210792
Mouse/Rat monoclonal anti-Ki-67 PE	eBioscience	Cat#12-5698-80<br>RRID:AB_11149672
Mouse monoclonal anti-MHC Class II (I-A/I-E) APC	eBioscience	Cat# 17-5321-81<br>RRID:AB_469454
Mouse monoclonal anti-MHC Class II (I-A/I-E) eFluor 450	eBioscience	Cat#48-5321-82<br>RRID:AB_1272204
Mouse monoclonal anti-CD11c BV605	BD Biosciences	Cat#563057
Mouse monoclonal anti-CD11c eFluor660	eBioscience	Cat#50-0114<br>RRID:AB_11151507
Mouse monoclonal anti-CD45R/B220 PerCP	eBioscience	Cat#561086<br>RRID:AB_2034009

Mouse monoclonal anti-CD11b APC-eFluor® 780	eBioscience	Cat#47-0112<br>RRID:AB_1603193
Mouse monoclonal anti-CD40 PE/Cy7	Biolegend	Cat#124621<br>RRID:AB_10933422
Mouse monoclonal anti-CD86 PE	eBioscience	Cat#12-0862-82<br>RRID:AB_465768
Mouse monoclonal anti-CD80 PE	eBioscience	Cat#12-0801-82<br>RRID:AB_465752
Mouse anti-Ea 52-68 peptide, biotin conjugated	eBioscience	Cat#13-5741-81<br>RRID:AB_657822
Streptavidin APC	eBioscience	Cat#17-4317-82
Mouse CD3 Functional Grade Purified	eBioscience	Cat#16-0032-86<br>RRID:AB_468853
Mouse CD28 Functional Grade Purified	eBioscience	Cat#16-0281-81<br>RRID:AB_468920
Phospho-Akt (Ser473) Antibody	Cell signaling Technology	Cat#9271<br>RRID:AB_329825
Rabbit Polyclonal IgG (H+L) Alexa Fluor647	Molecular Probes	Cat#A31573<br>RRID:AB_2536183
Mouse CD8 depletion monoclonal antibody, clone 53-6.7	eBioscience	Cat#14-0081-82<br>RRID:AB_467087
Anti-Apolipoprotein E antibody	abcam	Cat#ab183597
Goat anti-rabbit IgG H&L (Alexa Fluor® 488)	abcam	Cat#ab150077<br>RRID:AB_2630356
Mouse monoclonal anti-IFNγ AlexaFluor647	BD Biosciences	Cat# 557735<br>RRID:AB_396843
Human monoclonal anti-CD4 APC	BD Biosciences	Cat#555349<br>RRID:AB_398593
Human monoclonal anti-CD25 PerCP Cyanine 5.5	eBioscience	Cat#45-0259-41<br>RRID:AB_10717820
Human monoclonal anti-CD127 PE	eBioscience	Cat#12-1278-42<br>RRID:AB_10717663

Human monoclonal anti-CD45RA FITC	BD Biosciences	Cat#555488<br>RRID:AB_395879
Human monoclonal anti-CD197 (CCR7) PE	BD Biosciences	Cat#552176<br>RRID:AB_394354
Human monoclonal anti-CD11c APC	BD Biosciences	Cat#340544<br>RRID:AB_400520
Human monoclonal anti-HLA-DR FITC	BD Biosciences	Cat#347363<br>RRID:AB_400291
Human monoclonal anti-CD80 FITC	BD Biosciences	Cat# 560926<br>RRID:AB_10565975

Supplementary Table 3. List of mRNA primers used in the paper.

TARGET	FW	REV
mRPL	5’- GCGCCTCAAGGTGTTGGAT - 3’	5’- GAGCAGCAGGGACCACCAT – 3’
mCD36	5’- TGGGAGTTGGCGAGAAAACC-3’	5’- CAGGACTGCACCAATAACAGC -3’
mLDLR	5’- GTGTGACCGTGAACATGACTGC -3’	5’- CACTCCCCACTGTGACACTTGA -3’
mABCA1	5’- GGTTTGGAGATGGTTATACAATAGTTGT -3’	5’- TTCCCGGAAACGCAA -3’
mABCG1	5’- TTCATCGTCCTGGGCATCTT -3’	5’- CGGATTTTGTATCTGAGGACGAA-3’
mHMGCoAR	5’- TGTGGTTTGTGAAGCCGTCAT -3’	5’- TCAACCATAGCTTCCGTAGTTGTC -3’
mDHCR24	5’- AGAACTACCTGAAGACAAACCG -3’	5’- GAAGAGGTAGCGGAAGATGG -3’
hβ-actin	5’- CTGGCTGCTGACCCGAGG -3’	5’- GAAGGTCTCAAACATGATCTGGGT-3’
hApoE	5’- TGGCTACCAACCCCATCATC -3’	5’- GCAGGACAGGAGAAGGATACTCA -3’
hCD36	5’- TGGGAGTTGGCGAGAAAACC -3’	5’- CAGGACTGCACCAATAACAGC -3’
hLDLR	5’- GTGTGACCGTGAACATGACTGC -3’	5’- CACTCCCCACTGTGACACTTGA -3’
hHMGCoAR	5’- TGTGGTTTGTGAAGCCGTCAT -3’	5’- TCAACCATAGCTTCCGTAGTTGTC -3’
hABCA1	5’- GGTTTGGAGATGGTTATACAATAGTTGT -3’	5’- TTCCCGGAAACGCAAGTC -3’
hABCG1	5'-TTCATCGTCCTGGGCATCTT-3'	5'-CGGATTTTGTATCTGAGGACGAA-3'
hTNFα	5’- GCCCCCAGAGGGAAGAGTTCCC -3’	5’- CAGCTCCACGCCATTGGCCA -3’
hIL-6	5’- TCCACAAGCGCCTTCGGTCC-3’	5’- TGTCTGTGTGGGGCGGCTACA -3’
hIL-10	5’- GTGATGCCCCAAGCTGAGA -3’	5’- TCCCCCAGGGAGTTCACA -3’
hIL-1β	5’- GATGAAGTGCTCCTTCCAGGACCT -3’	5’- TGCTGTGAGTCCCGGAGCGT -3’
